# System Consolidation of Spatial Memories in Mice: Effects of Enriched Environment

**DOI:** 10.1155/2013/956312

**Published:** 2013-07-07

**Authors:** Joyce Bonaccorsi, Simona Cintoli, Rosa Mastrogiacomo, Sigrid Baldanzi, Chiara Braschi, Tommaso Pizzorusso, Maria Cristina Cenni, Nicoletta Berardi

**Affiliations:** ^1^Scuola Normale Superiore, Piazza dei Cavalieri 7, 56100 Pisa, Italy; ^2^Institute of Neuroscience of the CNR, 56124 Pisa, Italy; ^3^Pisa University, 56100 Pisa, Italy; ^4^Department of Neuroscience, Psychology, Drug Research and Child Health, NEUROFARBA, Università di Firenze, Viale Pieraccini, 50100 Firenze, Italy

## Abstract

Environmental enrichment (EE) is known to enhance learning and memory. Declarative memories are thought to undergo a first rapid and local consolidation process, followed by a prolonged process of system consolidation, which consist in a time-dependent gradual reorganization of brain regions supporting remote memory storage and crucial for the formation of enduring memories. At present, it is not known whether EE can affect the process of declarative memory system consolidation. We characterized the time course of hippocampal and cortical activation following recall of progressively more remote spatial memories. Wild-type mice either exposed to EE for 40 days or left in standard environment were subjected to spatial learning in the Morris water maze and to the probe test 1, 10, 20, 30, and 50 days after learning. Following the probe test, regional expression of the inducible immediate early gene c-Fos was mapped by immunohistochemistry, as an indicator of neuronal activity. We found that activation of the medial prefrontal cortex (mPFC), suggested to have a privileged role in processing remote spatial memories, was evident at shorter time intervals after learning in EE mice; in addition, EE induced the progressive activation of a distributed cortical network not activated in non-EE mice. This suggests that EE not only accelerates the process of mPFC recruitment but also recruits additional cortical areas into the network supporting remote spatial memories.

## 1. Introduction

Environmental enrichment (EE) is an experimental protocol classically defined as “a combination of complex inanimate and social stimulation” [1] and which provides animals with the opportunity to attain high levels of voluntary physical activity on running wheels and to enhance exploration, cognitive activity, and social interaction. Several studies point out that animals reared in EE show marked brain changes at functional, anatomical, and molecular levels [2–13] and in particular changes in plasticity factors and mechanisms [14, 15]. EE can indeed be used as a noninvasive strategy to modulate brain plasticity throughout life; EE can accelerate the development of the central nervous system [16–18], can reopen plasticity windows in the adult cortex [19, 20] and causes a significant improvement in learning and memory [14, 21–23], especially evident in aged animals [22, 24–32], or in animal models of neurodegenerative diseases [33–35].

Declarative memories depend initially on the medial temporal lobe system, including the hippocampus, but, over days to weeks, as these memories mature, they become increasingly dependent on other brain regions such as the neocortex [36–39]. This process of time-dependent gradual reorganization of the brain regions that supports remote memory storage and underlies the formation of enduring memories, is known as system-level memory consolidation or system consolidation [40, 41]. It has been demonstrated that the progressive stabilization of long-lasting memories is due to the reactivation of hippocampal-cortical connections [42, 43] and the strengthening of corticocortical connections, involving cortical plasticity mechanisms [41, 44–48]. Despite all the evidences showing that EE enhances cortical plasticity and learning and memory, there is no evidence on whether and how EE could affect the process of time-dependent system consolidation.

This study aimed at testing whether EE could affect the system-level memory consolidation process using a spatial memory paradigm and characterizing the time course of hippocampal and of cortical activation following recall of progressively more remote memories. 

Spatial memory is a declarative type of memory. The hippocampus plays an essential role in the formation of spatial memories [49–53]; subsequently, spatial memories become, in a gradual manner, additionally dependent on other cortical regions. Many studies point out that the privileged final storage site for remote spatial memories is the prefrontal cortex (PFC) and in particular the medial PFC (mPFC), including the anterior cingulate, prelimbic, and infralimbic cortices [47, 48, 54, 55]. Recently, however, it has been suggested that remote memories recall involves the coordinated activation of a broader network of cortical brain regions [56–58]. We have therefore characterized the time course of activation of a number of cortical areas in addition to the mPFC. We found that EE not only induces an earlier recruitment of mPFC, but also induces the progressive activation of a distributed cortical network that is not activated in standard housed mice.

## 2. Materials and Methods

### 2.1. Animal Treatment

Male and female C57BL/6 mice of 2 months of age were used in this study. All the procedures were approved by the Italian Ministry of Health. The animals were housed in an animal room with a 12 h/12 h light/dark cycle, with food and water available ad libitum. At 2 months of age, the animals were assigned to one of the following rearing conditions for 40 days: environmental enrichment (EE, *n* = 24) or standard condition (SC, *n* = 24). The SC rearing condition consisted of a 26 × 18 × 18 cm cage housing 3 animals. The EE rearing condition was achieved using a large cage (44 × 62 × 28 cm) containing several food hoppers, one running wheel for voluntary physical exercise, and differently shaped objects (tunnels, toys, shelters, stairs) that were repositioned twice a week and completely substituted with others once a week [33]. Two additional groups of control animals, age and gender matched to SC and EE groups, were housed in home cage standard condition (HC-SC, *n* = 8) or in home cage enriched condition (HC-EE, *n* = 7), and they did not perform any behavioural task.

### 2.2. Morris Water Maze (MWM)

The hidden platform version of the MWM test was performed [59]. A large water tank of 120 cm of diameter was filled with white opaque water at 22°C. An escape platform of 11 cm of side was submerged 1 cm below the water surface and placed in the center of the SW quadrant. The platform was maintained in this position for all the swim trials through the test. Mice were trained to swim to the platform in 4 daily trials, starting in pseudorandomly varied locations, with a 30 min interval, during 7 consecutive days. The trial was complete once the mouse found the platform or 60 sec had elapsed. If the mouse failed to find the platform on a given trial, the experimenter guided the mouse onto the platform. Once reaching the platform, each mouse was allowed to rest for 20 s on it. After each trial each mouse was returned to its home cage where it rested until the next trial. After the completion of training, spatial memory was assessed in a probe test; a recall probe trial was performed after 1, 10, 20, 30, and 50 days after the end of learning. We used an automated tracking system (Noldus Ethovision XT) for recording behavioural data from training and probe tests. For each trial we measured the latency (in sec) to reach the platform, the total distance (in cm) swam in order to reach the platform, and the average swim speed (in cm/s). For each probe trial we measured the amount of time spent in the target zone (23 cm in radius, centered on the location of the platform during training) and the average time spent in three other equivalent zones in each quadrant [55, 60].

### 2.3. Immunohistochemistry

Mice were anaesthetized and perfused via intracardiac infusion with 0.1 M PBS and then 4% paraformaldehyde (PFA, dissolved in 0.1 M phosphate buffer, pH 7.4) 90 min after the completion of behavioral testing. Brains were removed, fixed overnight in PFA, and then transferred to 30% sucrose solution and stored at 4°C. Coronal sections were cut at 40 *μ*m thickness on a freezing microtome (Sliding Leica microtome SM2010R, Leica Microsystems), and free-floating sections were prepared for immunohistochemistry. After a blocking step in 10% NGS and 0.5% Triton X-100 in PBS, sections were incubated in a solution containing 1% NGS, 0.3% Triton X-100, and anti-c-Fos primary rabbit polyclonal antibody (1 : 3000 rabbit anti c-Fos polyclonal antibody, Calbiochem, USA) for 36 h at 4°C. Subsequently, sections were transferred in a solution containing 1% NGS, 0.1% Triton X-100, and 1 : 200 anti-rabbit biotinylated secondary antibody (Vector Labs) in PBS. This was followed by incubation in ABC kit (Vector Labs) and final detection with DAB reaction kit (Vector Labs). Sections were finally mounted on gelatinized slides, dehydrated, and sealed with DPX mounting medium (VWR International, UK).

### 2.4. Analysis of c-Fos Positive Cells

Counting of c-Fos positive cells in different brain areas was performed using a CCD camera (MBF Bioscience, Germany) mounted on a Zeiss Axioskop (Zeiss, Germany) microscope and the Stereo- Investigator software (MBF Bioscience). Brain structures were anatomically defined according to a mouse brain atlas (Paxinos and Franklin [61]), and the regions of interest selected for measurement of c-Fos-positive nuclei were (numbers indicate the distance in millimeters of the sections from bregma) infralimbic cortex (IL, +1.94 mm); secondary motor cortex (M2, +0.98 mm); anterior cingulate cortex, area 1 and area 2 (aCC, +0.98 mm); dentate gyrus (DG, −1.94 mm); CA1 field of dorsal hippocampus (dCA1, −1.94 mm); CA3 field of dorsal hippocampus (dCA3, −1.94 mm); posterior parietal association cortex (pPtA, −1.94 mm); primary auditory cortex (Au1, −3.16 mm); primary visual cortex (V1, −4.16 mm); medial entorhinal cortex (MEnt, −4.16 mm). The number of c-Fos-positive cells was counted at 20x magnification, following a “blind procedure”, in 10–40 fields (50 × 50 *μ*m or 100 × 100 *μ*m) per section according to the size of brain structure and their density calculated (cells/mm^2^), using at least 5 sections for each structure.

### 2.5. Statistics

All results were expressed as mean ± s.e.m., and all statistical analysis were performed using statistical software package SigmaStat. For MWM performance in the learning phase, a two-way analysis of variance (ANOVA) for repeated measures (RM) was performed, considering both factor housing condition (EE or SC) and factor learning day, with post hoc analysis Holm-Sidak method. Performance in each probe test was compared with one-way ANOVA across circular zones (target zone versus the average of other zones) for each housing condition. The c-Fos protein expression in each area was analyzed with a two-way ANOVA for housing condition factor and probe test day factor, with post hoc analysis Holm-Sidak method.

## 3. Results

### 3.1. Hippocampus Is Activated following Spatial Memory Recall at All Temporal Points Tested Both in EE and SC Mice

**Figure 1:**
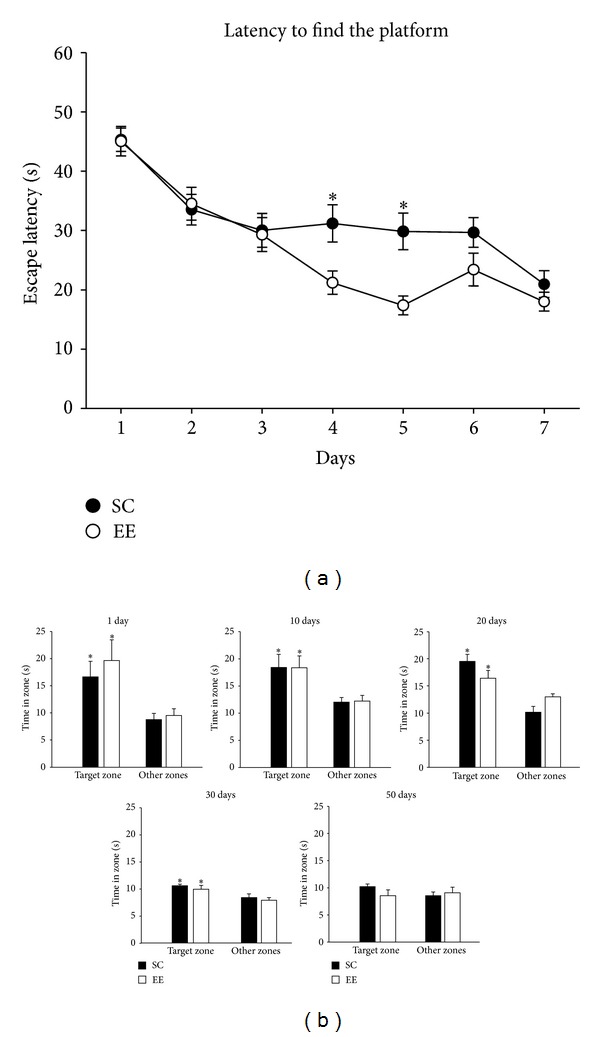
(a) Performances of SC (*n* = 24) and EE (*n* = 24) mice in the MWM. There is a significant learning effect (two-way RM ANOVA, *P* = 0.016), and a housing condition × day interaction (two-way RM ANOVA post hoc analysis Holm-Sidak method, for day 4, *P* = 0.005; for day 5, *P* = 0.001). * = statistical significance; error bars = s.e.m. (b) Evaluation of spatial memory for SC (1 day *n* = 5; 10 days *n* = 5; 20 days *n* = 5; 30 *n* = 5, 50 *n* = 4) and EE (1 day *n* = 5; 10 days *n* = 5; 20 days *n* = 5; 30 *n* = 5; 50 *n* = 4) mice. Time spent in the target zone (*T*), where the platform was placed, versus other equivalent zones (*O*), for recall probe tests. One-way ANOVA, post hoc analysis Holm-Sidak method, *1 day probe test*: in SC group, *T* versus *O*,  *P* = 0.044; in EE group, *T* versus *O*,  *P* = 0.036; *10 days probe test*: in SC group, *T* versus *O*,  *P* = 0.037; in EE group, *T* versus *O*,  *P* = 0.043; *20 days probe test*: in SC group, *T* versus *O*,  *P* < 0.01; in EE group, *T* versus *O*,  *P* = 0.048; *30 days probe test*: in SC group, *T* versus *O*,  *P* = 0.025; in EE group, *T* versus *O*,  *P* = 0.048; *50 days probe test*: in SC group, *T* versus *O*,  *P* = 0.070; in EE group, *T* versus *O*,  *P* = 0.226. * = statistical significance; error bars = s.e.m.

To test whether EE can affect the system consolidation process, we trained C57BL/6 mice, housed in different conditions (standard condition, SC *n* = 24 or environmental enrichment EE *n* = 24), in a spatial learning task, using the Morris water maze, and we analyzed the following parameters referred to the average of 4 daily trials, during 7 consecutive days: latency to find the platform (s), total distance swam (cm), and mean swim speed (cm/s). For the distance swam and the latency to reach the platform during acquisition, a significant learning effect for both housing conditions was found, but not a significant difference between the two groups (two-way RM ANOVA, for latency *P* = 0.016; for distance swan *P* < 0.001). For the latency parameter only, we found a housing condition × day interaction: multiple comparisons showed that the main differences resided on days 4 and 5 (Figure 1(a)) (Two-way RM ANOVA, post hoc analysis Holm-Sidak method, for day 4 *P* = 0.005; for day 5 *P* = 0.001). We also measured the mean swim velocity throughout the test, in order to exclude differences in navigation speed (data not shown): we observed a significant decrease in the mean swim velocity through the test (two-way RM ANOVA, *P* < 0.001), but neither a difference between housing condition (*P* = 0.276) nor a housing condition × day interaction (*P* = 0.163). 

Spatial memory was evaluated in a probe test in which the hidden platform was removed. We performed recall probe tests at 1, 10, 20, 30, and 50 days, and we quantified exploration in the target zone, a circular zone (radius: 23 cm) in quadrant where the platform was placed during training, and the average time spent in three other equivalent zones in each quadrant, for SC and EE mice (Figure 1(b)) [55, 60]. We found a significant difference between target zone versus the others in probe tests at 1, 10, 20, and 30 days, for both groups (see Figure 1(b) for details). 

After the probe test, mice were sacrificed and the protein c-Fos was immunolabeled as an indicator of neuronal activity. c-Fos expression was calculated as the density of number of c-Fos-positive cells in mm^2^. First we investigated c-Fos expression in the hippocampus, the structure that is responsible for the formation of long term spatial memory [49–53].

Levels of c-Fos expression in the hippocampus of control mice (Home cage mice, HC-EE and HC-SC mice) did not differ between housing conditions (HC-SC *n* = 8; HC-EE *n* = 7; one-way ANOVA, *P* = 0.736); levels of c-Fos protein for EE and SC mice were significantly greater than those in their home-cage controls at all retention intervals, (two-way ANOVA, post hoc analysis Holm-Sidak method, all *P* values <0.05), suggesting that the hippocampus is involved both in the formation and delayed recall of the spatial memory. We found a similar c-Fos expression in the EE and SC mice in all probe tests (two-way ANOVA, post hoc analysis Holm-Sidak method for housing condition, *P* = 0.731).

**Figure 2:**
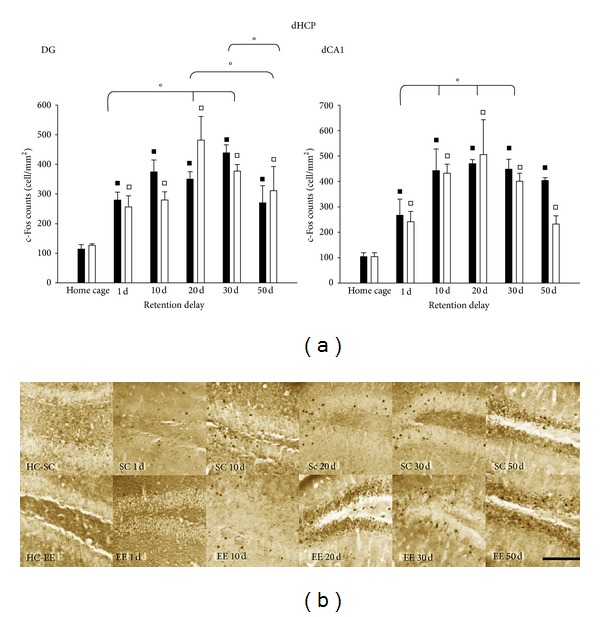
(a) c-Fos expression in subregions of dHPC for EE (open columns) and SC (filled columns) mice subjected to recall probe tests at 1, 10, 20, 30, and 50 days. *DG*: two-way ANOVA post hoc analysis Holm-Sidak method, SC versus EE, *P* = 0.968; HC-SC versus HC-EE, *P* = 0.503; SC versus HC-SC and EE versus HC-EE *P* < 0.05 for all retention intervals. Statistical differences for factor day were found between 1 and 20 days, 1 and 30 days, and 50 days versus 20 and 30 days, all *P* < 0.05. *dCA1*: two-way ANOVA post hoc analysis Holm-Sidak method, SC versus EE, *P* = 0.242; HC-SC versus HC-EE *P* = 0.979. Statistical difference factor day were found between 1 and 10, 20 and 30 days, all *P* < 0.05. ^*▪*^Statistical significance between EE and HC-EE; ^□^statistical significance between SC and HC-SC; °statistical significance for factor day; error bars = s.e.m. (b) Representative panel of c-Fos protein expression in DG for EE and SC animals, for all recall probe tests; scale bar 100 *μ*m.

We then focused on the dorsal hippocampus (dHCP), known to be specifically involved in spatial memory [60]; again, we observed the same c-Fos expression pattern in EE and SC group; activation increased with increasing retention interval up to 30 days (see Figure 2 for details).

### 3.2. EE Induces an Early Recruitment of the MPFC

**Figure 3:**
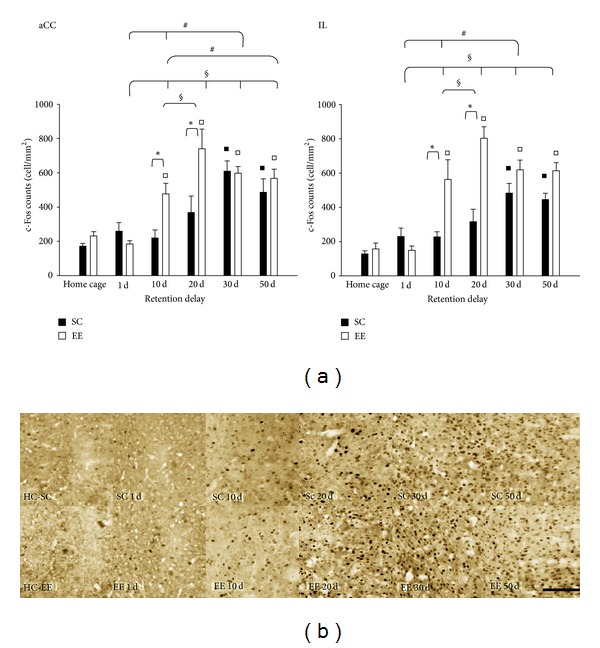
(a) c-Fos protein expression in aCC and IL. *aCC*: two-way ANOVA post hoc analysis Holm-Sidak method, HC-SC versus HC-EE *P* = 0.384; statistical significance for EE versus HC-EE at 10, 20, 30, and 50 days, and for SC versus HC-SC at 30 and 50 days, all *P* values < 0.05. Statistical difference between EE and SC at 1 day, *P* = 0.376; at 10 and 20 days, *P* < 0.01; at 30 days, *P* = 0.899; at 50 days, *P* = 0.383. Statistical difference within EE group were found between 10 and 20 days, and 1 day versus 10, 20, 30, and 50 days, all *P* < 0.05. Statistical difference within SC group were found between 1 and 30 days, and 10 day versus 30 and 50 days, all *P* < 0.05. *IL*: two-way ANOVA post hoc analysis Holm-Sidak method, HC-SC versus HC-EE, *P* = 0.451; statistical significance for EE versus HC-EE at 10, 20, 30, and 50 days, and for SC versus HC-SC at 30 and 50 days, all *P* values < 0.05. Statistical differences between EE and SC at 1 day, *P* = 0.300; at 10 and 20 days, *P* < 0.01; at 30 days, *P* = 0.084; at 50 days, *P* = 0.055. Statistical differences within EE group were found between 10 and 20 days, and 1 day versus 10, 20, 30, and 50 days, all *P* < 0.05. Statistical difference within SC group were found between 1 and 30 days, and 10 day and 30 days, all *P* < 0.05. ^*▪*^Statistical significance between EE and HC-EE; ^□^statistical significance between SC and HC-SC; *statistical significance between EE and SC; ^§^ = statistical significance for factor day within EE group; ^#^ = statistical significance for factor day within SC group; error bars = s.e.m. (b) Representative panel of c-Fos protein expression in aCC for EE and SC animals, for all recall probe tests; scale bar 100 *μ*m.

The results for c-Fos expression in the mPFC, the final memory storage site in the cortex, show that both the aCC and the IL have the same time course of c-Fos protein expression pattern; c-fos expression at 1 day did not differ from that in home cage animals, both for EE and SC mice, and there was no difference between EE and SC or HC-EE and HC-SC mice (two-way ANOVA, post hoc analysis Holm-Sidak method, all *P* values >0.05); however, starting from the probe test at 10 days, c-Fos expression was greater in EE group than HC-EE and SC groups, with a further increase at 20 days (see Figure 3 for details); only for EE animals did the c-Fos protein expression differ from that of HC control animals. For the probe tests at 30 and 50 days we found that the c-Fos expression in SC animals differed from that of HC-SC animals; values of SC and EE groups did not differ (two-way ANOVA, post hoc analysis Holm-Sidak method; see Figure 3 for details). 

### 3.3. EE Induces the Involvement of Distributed Cortical Network in Supporting Remote Spatial Memory

**Figure 4:**
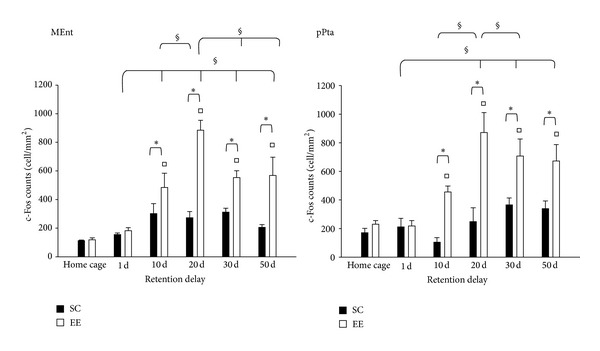
c-Fos protein expression in MEnt and pPta. *MEnt*: two-way ANOVA post hoc analysis Holm-Sidak method, HC-SC versus HC-EE *P* = 0.914; statistical significance for EE versus HC-EE at 10, 20, 30 and 50 days. Statistical difference between EE and SC at 1 day, *P* = 0.782; at 10, 20, 30 and 50 days, *P* < 0.01. Statistical difference within EE group were found between 1 day versus 10, 20, 30 and 50 days, and 20 days versus 10, 30 and 50 days, all *P* values < 0.05. *pPta*: two-way ANOVA post hoc analysis Holm-Sidak method, HC-SC versus HC-EE *P* = 0.497; statistical significance for EE versus HC-EE at 10, 20, 30 and 50 days, all *P* values < 0.05. Statistical difference between EE and SC at 1 day, *P* = 0.300; at 10 and 20 days, *P* < 0.01; at 30 days, *P* = 0.084; at 50 days, *P* = 0.055. Statistical difference within EE group were found between 1 day versus 20, 30 and 50 days, and 20 day versus 10 and 30 days, all *P* values < 0.05. Statistical difference within SC group were found between 1 and 30 days, and 10 day and 30 days, all *P*  values < 0.05. ^*▪*^Statistical significance between EE and HC-EE; ^□^statistical significance between SC and HC-SC; *statistical  significance between EE and SC; ^§^ = statistical significance for factor day within EE group; error bars = s.e.m.

**Figure 5:**
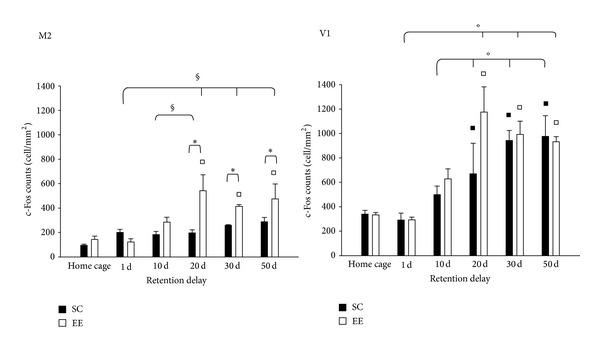
c-Fos protein expression in V1 and M2. *M2*: two-way ANOVA post hoc analysis Holm-Sidak method, HC-SC versus HC-EE, *P* = 0.416; statistical significance for EE versus HC-EE at 20, 30, and 50 days. Statistical difference between EE and SC at 1 day, *P* = 0.288; at 10 days, *P* = 0.154; at 20, 30, and 50 days, *P* < 0.05. Statistical differences within EE group were found between 10 and 20 days, and 1 day versus 20, 30, and 50 days, all *P* values < 0.05. *V1*: two-way ANOVA post hoc analysis Holm-Sidak method, HC-SC versus HC-EE *P* = 0.907; statistical significance for EE versus HC-EE at 20, 30, and 50 days, and for SC versus HC-SC at 20, 30, and 50 days, all *P* values < 0.05. Statistical significance for factor day was found between 1 day versus 20, 30, and 50 days, and 10 days versus 20, 30, and 50 days, all *P* values < 0.05. ^*▪*^Statistical significance between EE and HC-EE; ^□^statistical significance between SC and HC-SC; * = statistical significance between EE and SC; °statistical significance for factor day ^§^ = statistical significance within EE group; error bars = s.e.m.

**Figure 6:**
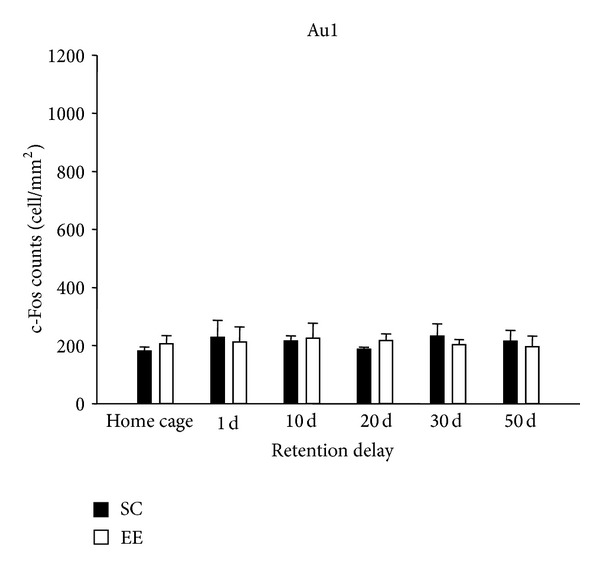
c-Fos protein expression in Au1. The data showed no difference between groups (two-way ANOVA, housing condition factor, *P* = 0.996, day of probe test factor, *P* = 0.943, housing condition × day of probe test interaction, *P* = 0.938). In all probe tests, EE and SC groups did not differ from their home cage controls.

To examine the time-dependent reorganization of neuronal activation in a brain-wide manner, we observed c-Fos protein expression in other cortical areas that are important for the construction of spatial maps. Using several techniques, Wang et al. [62] determined that distinct areas of extrastriate visual cortex are gateways for ventral and dorsal streams in the mouse. The dorsal stream includes the network hippocampus—medial entorhinal cortex [63]—posterior parietal cortex [64] for spatial navigation; in addition, the dorsal stream is connected to auditory cortex and to frontal areas, such as cingulate cortex, infralimbic cortex, and motor areas [62]. First we investigated c-Fos expression in MEnt and in pPta, and we found that activation at 1 day in both areas was similar in EE mice, SC mice, and did not differ from that in their home-cage controls (two-way ANOVA, post hoc analysis Holm-Sidak method, all *P* values > 0.05); however, in the other probe tests performed, significant differences between EE and SC mice and between EE and HC-EE mice were found (see Figure 4 for details). Then, we observed c-Fos expression in V1 and M2, for they are connected to the dorsal stream and there is a direct monosynaptic connection between motor and visual cortices [65]. We found that EE group was statistically different from SC and HC-EE groups in M2, only in probe test performed at 20, 30, and 50 days; instead, in V1, we did not find any difference between EE and SC group, only an increase in c-Fos expression for the late retention delays in both groups (see Figure 5 for details). Finally, we investigated c-Fos expression in Au1, a sensory cortex not supposed to be involved in spatial learning, and we demonstrated that activation in this area was similar in all groups (see Figure 6 for details).

## 4. Discussion

In this study, we provide the first evidence that EE can affect the time-dependent spatial memory system consolidation. C57BL/6 mice, housed in standard or in enriched condition, were subjected to spatial learning and then tested up to 50 days after learning to evaluate consolidation process. Using the expression of c-Fos protein as an indicator of neuronal activity in a brain-wide manner we have found indications for a difference both in the time course and in the network of cortical areas recruited for recent and remote recall between EE and non-EE animals. 

In agreement with previous studies [47, 55] there was a progressive increase in c-Fos protein expression in both aCC and IL, as consolidation process proceeded. We showed that EE induces an earlier recruitment of aCC and IL with respect to SC mice; these areas were recruited following recall of spatial memory in the EE group as early as 10 days after learning, while they were recruited only 30 days after learning in SC mice. The final storage site in the cortex could be the aCC, while the IL could correlate with motivational aspects of performance, encoding other significant aspects of the environment, such as salient landmarks or preferred locations [66]. The aCC was found to be activated after remote memory recall in a number of tasks [47, 48, 54, 55], and, conversely, inactivation of the aCC disrupted recall of remote five-arm discrimination [47], contextual fear [48], and MWM [55] memories. The aCC is highly interconnected to other prefrontal regions and is reciprocally connected to sensory, motor, and limbic cortices [67, 68]; therefore, this connectivity places the aCC in favorable position, raising the possibility that this region coordinates retrieval of remote memories stored in distributed cortical networks. The earlier recruitment of aCC and IL in EE animals could imply an earlier independence of spatial memory recall from hippocampal activation in EE animals. Indeed, in animals provided with running wheels, a component of EE, block of hippocampal activation ceased to block recall of contextual fear memory at shorter time distance from learning with respect to sedentary animals [69]. 

In addition, we demonstrated that EE induces the involvement of a distributed cortical network in supporting remote spatial memory which is not activated in non EE animals. We observed that MEnt and pPta were activated following memory recall at 10 days in EE group. Both areas were included in the dorsal network for spatial navigation [60]; the entorhinal cortex contains a spatial representation of environment and plays an interface role between the hippocampus and neocortex [70]; instead, the parietal cortex, specifically the multisensory posterior region, translates coordinate information from spatial maps in the entorhinal cortex and hippocampus into egocentric representations [59, 71]. We also investigated c-Fos protein expression in V1 and M2, and we found that EE group showed a greater activation in M2 than SC group, for probe test performed at 20, 30, and 50 days. In V1, instead, we did not find any difference between EE and SC group, only an increase in c-Fos expression for the late retention delays in both groups; a recent study [58] showed that V1 could belong to the network of fear contextual memory, although its activation did not change between recent and remote memories. In our study, the V1 pattern activation could be induced by its engagement in attentional process on account of the spatial complex task. The involvement of M2 and V1 is not surprising since they are connected to the dorsal stream [62], and a direct monosynaptic connection between motor and visual cortices was identified [65]. 

For the hippocampus we found no difference between EE and SC animals. Our results are consistent with the idea that hippocampus is responsible for encoding spatial memory [49–53]; its activation in remote spatial memory recall is not in agreement with studies that showed a progressive reduction in hippocampus activation with increasing retention interval [47, 48, 54], though it is in line with the hypothesis that remote memory never becomes totally independent from the hippocampus [72]. In a more recent study, indeed, Lopez et al. [73] demonstrated that hippocampus recruitment in the recall of remote memories was influenced by the environmental conditions, such as cue saliency and complexity of the task in which memories are initially formed and subsequently recalled; thus the rich spatial details and the complexity of the training in MWM could account for the hippocampal activation found also for remote memory recall. Moreover, it has been found that that precise real-time inhibition of the dorsal CA1 region, using optogenetic method, was sufficient to impair remote recall [74].

We found that EE mice were faster, considering latency parameter, in learning the position of the target platform in comparison to SC mice, but there was no significant difference between groups in the probe tests. These results are consistent with other studies in the literature which used, in rodents living in EE or provided with running wheels, intensive learning protocol for the MWM such as that used by us (4 daily trials for 7 consecutive days) [21]. We decided to use a behavioural protocol that maximizes the results of spatial learning task because our purpose was to examine possible differences between EE and SC groups in the time course of system consolidation without confounding effects due to differences in the results of learning, that is in the probe test, and also to be confident in the formation of a remote memory. The results of c-Fos data are an indication that the same result (a successful probe test) can be obtained through a different balance of hippocampal and cortical activation during system consolidation. Also in the Maviel and Bontempi paper [47] response accuracy in animals tested on either day 1 or 30 was similar while cortical activation strongly differed. The faster recruitment of cortical areas found in EE animals and the activation of a distributed cortical network, involving prefrontal and other associative cortices, activated in EE but not in SC animals, could suggest that the quality of the recalled memory is different in the two groups of animals; indeed, activation of prefrontal cortex has been correlated with development of the ability to create a memory that is vivid and rich in contextual details in humans [75] and activation of associative sensory cortices supports memory storage and retrieval of sensory stimuli that have acquired a behavioral salience with the experience [56].

How could EE act on the recruitment of cortical networks during system consolidation? One possibility is via its well-known action on hippocampal neurogenesis. It has been proposed that new neurons generated in the DG become functionally integrated into existing neural circuits [76]; in fact the spatial training when new neurons are more receptive to surrounding neuronal activity favored their subsequent recruitment upon remote memory retrieval [77, 78]. Thus, these tagged adult-generated neurons, once mature, are recruited into hippocampal networks underlying remote spatial memory representation. Therefore tampering with the level of hippocampal neurogenesis could interfere in the hippocampus-only dependent period of memory. Indeed, it has been demonstrated that new hippocampal neurons were recruited into neuronal networks supporting retrieval of remote spatial memory and that the enhanced neurogenesis by voluntary running-wheel exercise sped up the disengaging from hippocampus [69]. Since EE was found to increase hippocampal neurogenesis and promote the survival of newly generated neurons [14], it is plausible that EE may accelerate the recruitment of extrahippocampal areas. In its turn, EE action on hippocampal neurogenesis is likely mediated by neurotrophins, such as brain-derived neurotrophic factor (BDNF), or by insulin growth factor-l (IGF-1) [9, 10], which affect hippocampal neurogenesis and hippocampal and cortical plasticity [14, 79, 80]. IGF-1 plays an important role in cell growth and development, and it also upregulates neurogenesis in the adult hippocampus [81–83]. In the adult brain, IGF-1 has been shown to mediate both the neuroprotective effects of physical exercise and the enhancement caused by exercise in hippocampal plasticity and in learning and memory [79]. Moreover IGF-1 mediates the increased expression of BDNF subsequent to EE and physical exercise [84–86]. BDNF has been shown to regulate adult hippocampal neurogenesis, to mediate EE effects on it, to modulate plasticity during learning by activating signaling pathways that modify local synaptic targets and have long-term effects on transcription, and to mediate the expression of hippocampal LTP, in both the early and late phases [80, 83, 87, 88]. 

Another nonexclusive possibility is that molecular mechanisms could “tag” the activated synapses in hippocampal and cortical networks at the time of memory encoding; this early tagging could guide the reactivation of proper hippocampal-cortical connections associated with the specific memory. A recent study showed indeed that cortical tagging seems to be highly specific for precise memory trace and the impairment of early cortical tagging tampers with the postacquisition hippocampal-cortical dialogue, preventing the formation of remote memory [41]. Moreover, they demonstrated that early tagging triggers specific signaling cascades, leading to histone-tail acetylation in the cortex and that the histone deacetylase inhibitor improves remote memory retrieval [41]. Thus, early tagging acts on epigenetic modification that could mediate remote memory formation and retrieval, so EE could modulate the long-term memory formation and consolidation through chromatin remodeling, such as histone-tail acetylation, known to be increased in EE.
